# Assessment of the efficacy of Ankaferd blood stopper on the prevention of postoperative pericardial adhesions

**DOI:** 10.5830/CVJA-2014-011

**Published:** 2014-06

**Authors:** Yunus Nazli, Necmettin Colak, Mehmet Fatih Alpay, Omer Nuri Aksoy, Ismail Olgun Akkaya, Omer Cakir, Hacer Haltas

**Affiliations:** Department of Cardiovascular Surgery, University of Turgut Ozal, Ankara, Turkey; Department of Cardiovascular Surgery, University of Turgut Ozal, Ankara, Turkey; Department of Cardiovascular Surgery, University of Turgut Ozal, Ankara, Turkey; Department of Cardiovascular Surgery, University of Turgut Ozal, Ankara, Turkey; Department of Cardiovascular Surgery, University of Turgut Ozal, Ankara, Turkey; Department of Cardiovascular Surgery, University of Turgut Ozal, Ankara, Turkey; Department of Pathology, University of Turgut Ozal, Ankara, Turkey

**Keywords:** cardiac operation, Ankaferd, pericardial adhesion

## Abstract

**Objectives:**

Ankaferd has been used as a blood-stopping agent and it may also have an anti-inflammatory effect. We investigated the efficacy of Ankaferd in preventing postoperative pericardial adhesions in an experimental rabbit model.

**Methods:**

Sixteen New Zealand white rabbits were used and categorised into two groups: an Ankaferd and a control group. The Ankaferd group of rabbits was treated with a sponge impregnated with Ankaferd solution, which was applied over the abraded epicardium. A sponge impregnated with 0.9% isotonic NaCl solution was applied to the control group using the same protocol. Scores for adhesion and visibility of coronary vessels were graded by macroscopic examination, and pericardial tissues were analysed microscopically in terms of inflammation and fibrosis.

**Results:**

In the Ankaferd group, the adhesion scores were significantly higher than in the control group (*p* = 0.007). When the groups were compared according to the prevalence of fibrosis and degree of inflammation, the Ankaferd group was found to be statistically significantly different from the control group in terms of prevalence of fibrosis (*p* = 0.028).

**Conclusion:**

Topical application of Ankaferd to prevent postoperative pericardial adhesions increased adhesion and fibrosis scores.

## Abstract

Postoperative pericardial adhesion formation occurs frequently after cardiac surgery and is an important cause of morbidity and mortality at the time of re-operation. As the number of patients undergoing cardiac surgery continues to increase, the number of potential candidates for re-operation is increasing exponentially. Despite ongoing improvements during cardiac re-operation surgery, the presence of pericardial adhesions not only adds to the surgery time but also increases the risk of life-threatening injuries to the heart, great vessels or previously placed coronary bypass conduits by obscuring the true anatomy.^1^

Various methods and materials have been investigated to prevent or reduce the severity of postoperative adhesions in the retrosternal spaces and mediastinal structures. Fibrinolytic agents, histamine antagonists, anti-coagulants, anti-inflammatory drugs (corticosteroids, non-steroidal drugs), antibiotics, several natural physical barriers (heterologous pericardium, omentum, peritoneum, amnion, fibrin, gelatin, collagen and hyaluronic acid) and synthetic physical barriers (rubber, silicon-based materials, cellulose, polytetrafluoroethylene, polyvinyl alcohol and polyester derivatives) have been tried with variable success for such purposes.^2^-^6^ Unfortunately, despite continuous advances and research, to date there is no ideal method to prevent or reduce postoperative pericardial adhesion formation.

Ankaferd blood stopper® (ABS) (Ankaferd Ilac Kozmetik AS, Istanbul, Turkey) is a folkloric medicinal plant extract. Ankaferd has been used as a blood-stopping agent against various types of bleeding. It has been approved by the Ministry of Health in Turkey for the management of bleeds due to external injury and dental surgery. It has also been used as a topical agent for the prevention of postoperative intra-abdominal fibrosis in experimental studies, and variable results have been obtained.^7^,^8^

To the best of our knowledge, there is no report on the effect of Ankaferd in preventing postoperative pericardial adhesions to date. The present experimental study was designed to investigate the effect of intrapericardially administered Ankaferd on reducing postoperative pericardial adhesion formation in the rabbit model.

## Methods

The study protocol was approved by the Ethics Committee for Animal Research, Ankara Education and Research Hospital, Ankara, Turkey. Sixteen New Zealand white rabbits weighing 2.5 to 3.0 kg were anesthetised with 35 mg/kg ketamine hydrocloride and 5 mg/kg xylazine administered intramuscularly.^9^,^10^ After disappearance of the pedal reflex in the hind limbs, the rabbits were placed in the supine position on a heated operating table and their temperature was maintained at 39°C by monitoring their rectal temperature.^11^,^12^ The pedal reflex was checked every five minutes throughout the surgical procedure.

A venous line was established in the ear and a saline solution was infused at a rate of 3 mg/kg/h. Prophylactic antibiotic (cefazolin sodium 40 mg/kg) was given intravenously just before the operation. All had continuous two-lead electrocardiograph monitoring during the surgery. A paediatric facial mask in which oxygen gas flowed at a rate of 200 ml/min was placed on each rabbit.^13^

The surgical procedure was performed sterilely. After a midline muscle and skin incision was made over the sternum, the xiphoid process was carefully detached from the sternal part of the diaphragm. A median sternotomy was then performed; the median incision went down from the xiphoid process towards the jugular notch of the sternum exactly along the midline of the sternum so that injury to the parietal pleura was avoided. Sternal retractors were used to spread the sternal edges and maintain surgical exposure. The epicardium and parietal pericardium related to the right ventricle atrium, and right and left ventricle were abraded with 10 vertically reciprocal movements of dry gauze in order to create local inflammation.^13^

The rabbits were divided into two groups: the Ankaferd group was treated with a sponge that had been soaked in a 2-ml concentration of ABS solution (Ankaferd blood stopper® ampoule, 2 ml, Istanbul, Turkey) and applied over the abraded epicardium for five minutes (*n* = 8). The sponge was then removed. The abraded areas of the epicardium were irrigated immediately with enough saline to dispose of the remaining ABS. In the control group, the sponge was soaked in a 0.9% isotonic NaCl solution (serum fizyolojik 0.9% NaCl, 5 ml/ampoule, Adeka, Turkey) and was applied to the surface of the abraded epicardium for five minutes (*n* = 8). The sponge was then removed. The investigators were blinded during the application of Ankaferd or saline.

The sternum was closed with three interrupted sutures using 3-0 nylon and a needle with a tapered point. The muscle layers and skin were then closed with continuous sutures using 4-0 nylon and a cutting needle. The rabbits were allowed to recover. During the surgical procedure, all rabbits exhibited spontaneous respiration and loss of the pedal reflex.

The rabbits were sacrificed two weeks after surgery with a lethal dose of pentobarbital (150 mg/kg) (Nembutol, IE Ulagay, Istanbul, Turkey). The heart and pericardium were removed *en bloc*. Specimens were fixed in 10% formaldehyde, embedded in paraffin and sectioned into 4-μm slices, which were stained with haematoxylin and eosin to assess the inflammatory reaction and degree of fibrosis, and to check for remnants of the pericardial substitute in the two groups.

## Macroscopic examination

The heart and pericardium were removed with the anterior chest wall *en bloc*. The severity of pericardial adhesions and visibility of coronary vessels were evaluated by the same two blinded observers and scored. The following qualitative grading system was used to evaluate the tenacity of the adhesions: 0 = no adhesions; 1 = mild adhesions (transparent filmy adhesions separable by lifting the pericardium from the myocardium without dissection); 2 = moderate adhesions (fibrous and easily separated by blunt dissection); 3 = severe adhesions (thick, requiring aggressive blunt dissection); 4 = very severe adhesions (multiple thick adhesions requiring aggressive dissection that damaged adherent tissue).^14^ In addition, another grading system was used to evaluate the visibility of the coronary arteries: 0 = clearly visible, 1 = blurred, 2 = completely obscured.^6^

## Light microscopic examination

After the macroscopic scoring, the paraffin-embedded heart tissues (segments of the pericardium and heart from the site of abrasion, which had been marked with a prolene stitch) were cut into 4-μm thick sections and stained with haematoxylin and eosin. Histopathological evaluation was performed by one pathologist who was blinded to the study groups.

The severity of the inflammatory reaction was based on quantification of the inflammatory cells (i.e. neutrophils, plasma cells, lymphocytes) and inflammatory foci. The scoring schemes of Lu *et al.*^15^ were used to grade inflammation (0 = no cell infiltration; 1 = sparse, focal infiltration of lymphocytes and plasma cells; 2 = focal infiltration of neutrophils, plasma cells and lymphocytes; and 3 = diffuse infiltration of neutrophils, plasma cells and lymphocytes), and fibrosis (0 = no fibrous reaction; 1 = sparse, focal fibrous connective tissue, hyalinisation and fibrin deposition; 2 = a thin layer of focal fibrous connective tissue, hyalinisation and fibrin deposition; and 3 = a thick layer of focal fibrous connective tissue, hyalinisation and fibrin deposition).

## Statistical analysis

The sample size of our study was calculated with G*Power (G*Power Ver. 3.00.10, Franz Faul, Üniversität Kiel, Germany, http://www.psycho.uniduesseldorf. de/aap/projects/gpower/) statistical packages. The required sample size for 80% power, α = 0.05 type I error, β = 0.20 type II error, and *f* = 0.70 effect size was calculated as 16, including eight New Zealand white rabbits in each group. To protect the study from potential loss to follow up, one more rabbit was included in each group and the study was completed with a sample size of 18.

Data coding and statistical analyses were conducted with SPSS (version 15; SPSS Inc, Chicago, IL, USA). Following the entering of the rabbits’ data into the computer, all the necessary diagnostic checks and corrections were performed. Conformity of the measured values to normal distribution was examined graphically and using a Shapiro-Wilks test.

In presenting descriptive statistics, numbers and percentages were used for categorical variables, and medians and ranges were used for non-normally distributed variables. The Mann-Whitney *U*-test was used to compare the median values of the groups. The chi-square test was performed to evaluate the difference in the proportion of rabbits in the groups. Groups that were close to each other were combined and a 2 × 2 table chi-square test was created. The likelihood ratio test was used for the comparison of these groups. The two-tailed test of *p* ≤ 0.05 was considered statistically significant.

## Results

All animals tolerated the procedure with no apparent postoperative complications. Results were analysed in terms of macro- and microscopic findings.

## Macroscopic findings

**Fig. 1.**
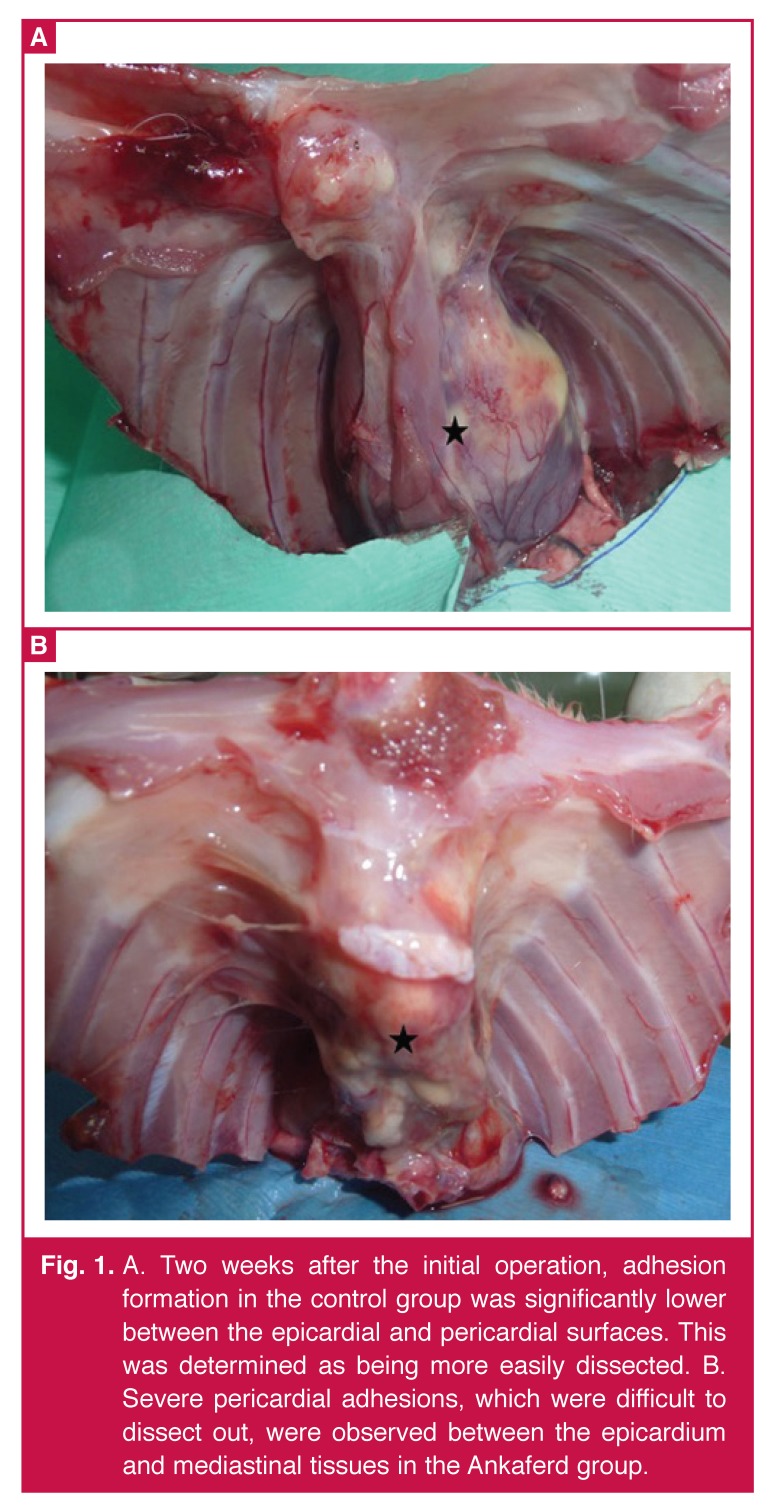
A. Two weeks after the initial operation, adhesion formation in the control group was significantly lower between the epicardial and pericardial surfaces. This was determined as being more easily dissected. B. Severe pericardial adhesions, which were difficult to dissect out, were observed between the epicardium and mediastinal tissues in the Ankaferd group.

A total of 16 rabbits were evaluated for grading of pericardial adhesions by macroscopic findings. In six cases in the control group and one case in the Ankaferd group, pericardial adhesions were split by blunt dissection (Fig. 1A). By contrast, seven of the Ankaferd group and two of the control group were associated with tight adherences to the sternum and the rest of the pericardium, requiring sharp dissection (Fig. 1B).

**Fig. 2.**
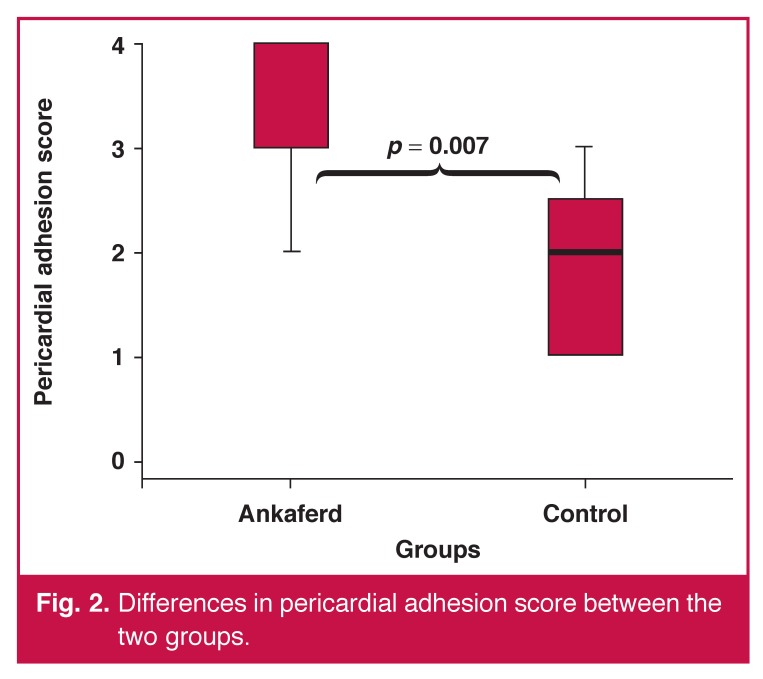
Differences in pericardial adhesion score between the two groups.

There were statistically significant differences between the Ankaferd and control groups in terms of the adhesion score [Ankaferd vs control group: 3 (2–4) vs 2 (1–3), *p* = 0.007] (Fig. 2). There was no statistically significant difference between the Ankaferd and control group in terms of the visibility of coronary vessels score [Ankaferd vs control group: 1 (1–2) vs 1 (1–2), *p* = 0.105] (Table 1). When the prevalence of pericardial adhesion was compared, there was a positive trend in the odds ratio for severe to very severe adhesion in the Ankaferd group (Ankaferd vs control group: 87.5 vs 25%, respectively) (Table 2). When the groups were compared according to the degree of pericardial adhesions and the visibility of coronary vessels score, there were statistically significant differences between the Ankaferd and control group (*p* = 0.009, *p* = 0.033, respectively) (Table 2).

**Table 1:** Results of macroscopic and microscopic scores between the control and Ankaferd groups.

*Variables*	*Ankaferd group (n = 8) Median (range)*	*Control group (n = 8) Median (range)*	*Z*	*p*
Macroscopic scores				
Pericardial adhesions	3 (2–4)	2 (1–3)	2.680	0.007
Visibility of coronary vessels	2 (1–2)	1 (1–2)	2.000	0.105 (NS)
Microscopic scores				
Inflammation	2 (2–3)	3 (1–3)	1.061	0.382 (NS)
Fibrosis	3 (3–3)	3 (1–3)	1.852	0.234 (NS)

NS, not statistically significant (*p* > 0.05).

**Table 2:** The distribution of the macroscopic and microscopic scores between the control and Ankaferd groups

*Variables*	*Ankaferd group n (%)*	*Control group n (%)*	*c^2^*	*p*
*Macroscopic scores*				
Pericardial adhesions				
Mild – moderate (1+2)	1 (12.5)	6 (75)	6.904	0.009
Severe – very severe (3+4)	7 (87.5)	2 (25)		
Visibility of coronary vessels				
Blurred (1)	1 (12.5)	5 (62.5)	4.557	0.033
Completely obscured (2)	7 (87.5)	3 (37.5)		
*Microscopic scores*				
Inflammation				
None – mild (0+1)	0 (0.0)	1 (12.5)	1.453	0.220 (NS)
Moderate – severe (2+3)				
Fibrosis	8 (100)	7 (87.5)		
Mild – moderate (1+2)	0 (0.0)	3 (37.5)	4.857	0.028
Severe (3)	8 (100)	5 (62.5)		

NS, not statistically significant (*p* > 0.05).

In our study, there was no infection or delayed healing at the wound site. Results of the macro- and microscopic scores and distribution of the scores between the control and Ankaferd groups are shown in Tables 1 and 2.

## Microscopic findings

**Fig. 3.**
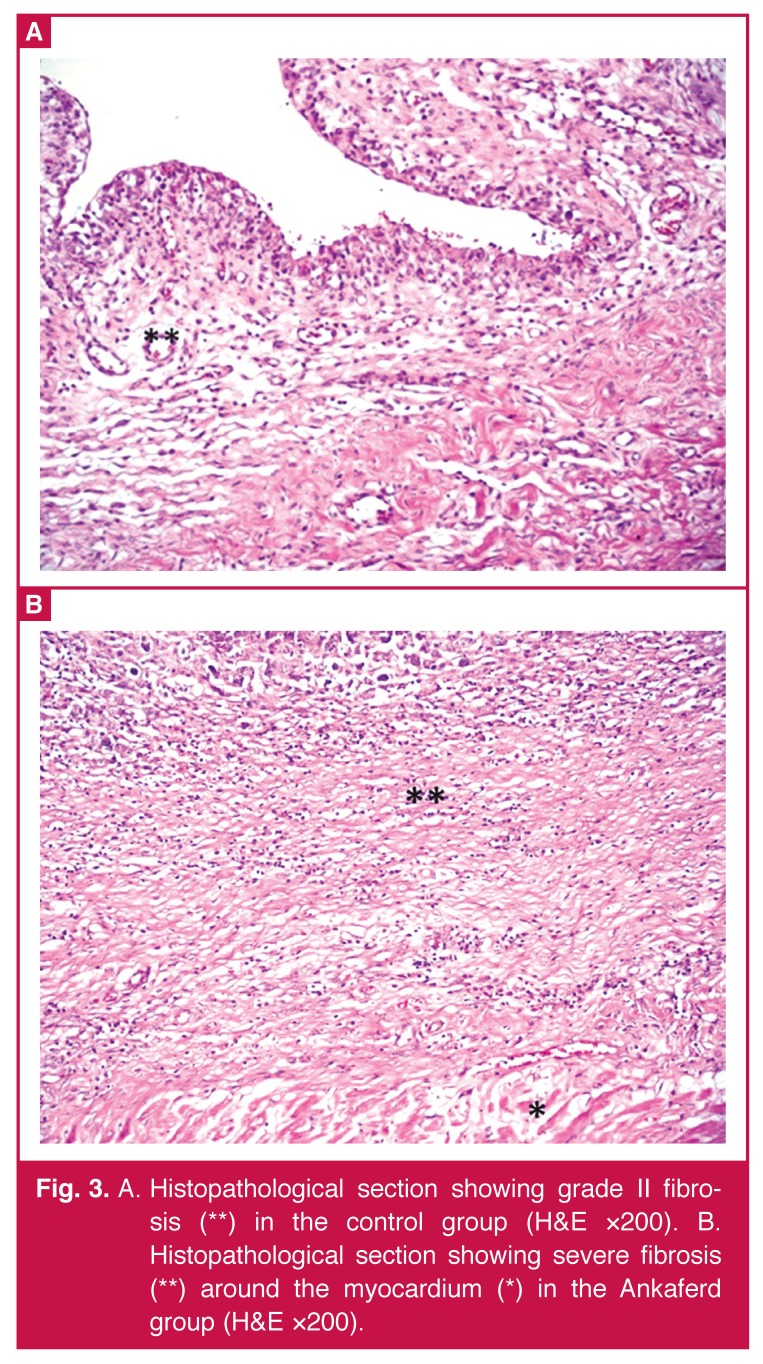
A. Histopathological section showing grade II fibrosis (**) in the control group (H&E ×200). B. Histopathological section showing severe fibrosis (**) around the myocardium (*) in the Ankaferd group (H&E ×200).

**Fig. 4.**
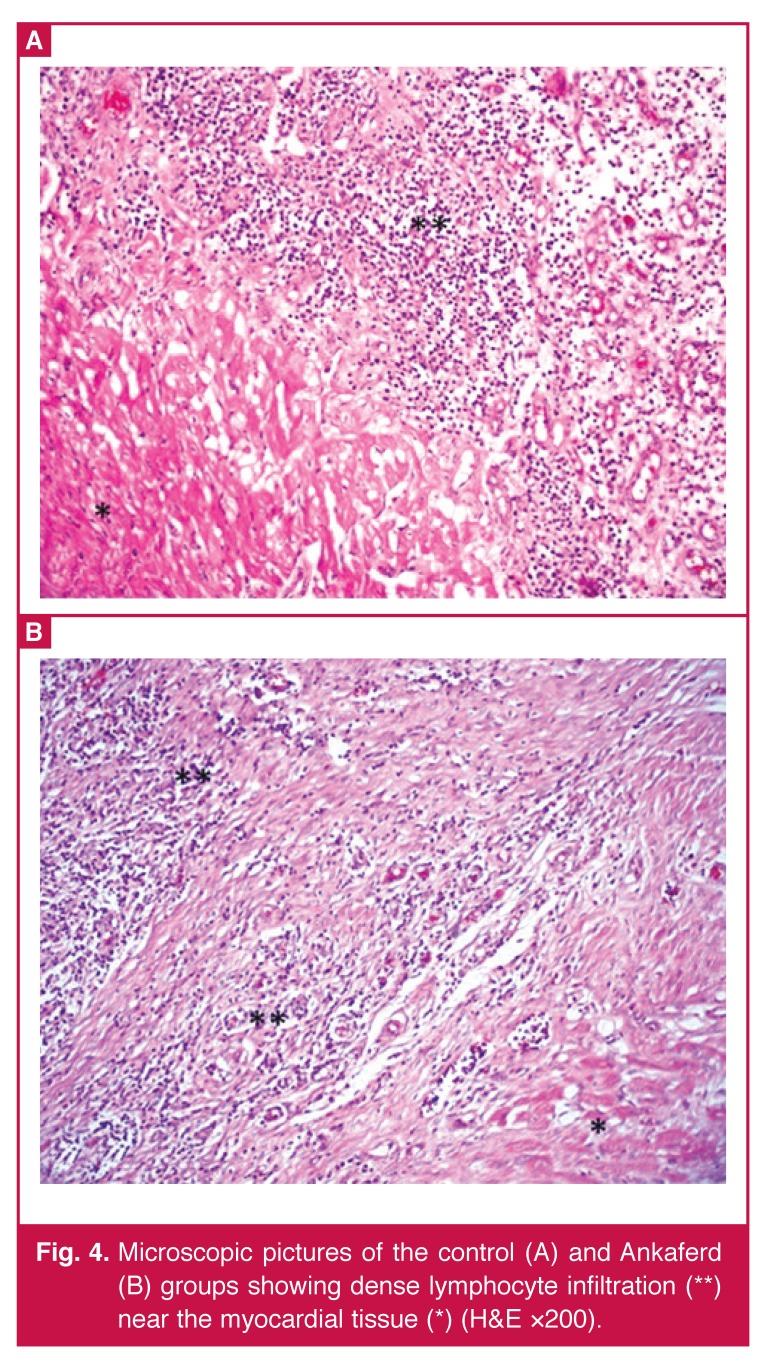
Microscopic pictures of the control (A) and Ankaferd (B) groups showing dense lymphocyte infiltration (**) near the myocardial tissue (*) (H&E ×200).

There were no statistically significant differences between the Ankaferd and control groups in terms of severity of fibrosis (*p* = 0.234) (Table 1). However, severe fibrosis was present in 100% of the Ankaferd group and 62.5% of the control group. When the groups were compared according to the prevalence of fibrosis and degree of inflammation, statistically significant differences between the groups were found in the Ankaferd group only in terms of the prevalence of fibrosis (*p* = 0.028) (Fig. 3A, B). There were no statistically significant differences between the Ankaferd and control group with regard to degree of inflammation (*p* = 0.220) (Fig. 4A, B) (Table 2).

## Discussion

One of the primary long-term postoperative concerns after a sternotomy is the formation of pericardial adhesions during the healing process. These adhesions are an important cause of morbidity and mortality in cardiac surgery.^16^ Pericardial adhesions may attach the heart to the undersurface of the sternum and neighboring structures, compromise right ventricular contraction, and restrict left ventricular diastolic filling. They can also obscure the anatomy of the heart, coronary arteries and great vessels, and coronary grafts if present, immensely complicating the procedure for re-operation by prolonging surgery time, and potentially escalating serious mediastinal injury during re-entry.^17^,^18^

Adhesion formation occurs as a consequence of the inflammatory response to surgical trauma, which can start within a few hours of surgery, as routine surgical procedures consist of tissue handling, including abrasion, desiccation, ischaemia, haemorrhage, exposure to foreign material and overheating by lamps.^18^ Histological examination of pericardial tissues from animals undergoing cardiac surgical procedures indicates that damage to the mesothelium (especially by abrasion) is adhesiogenic.^19^,^20^

It is known that mesothelial cells are responsible for the fibrinolytic properties of coelomic cavities, and damage to these cells is the trigger for adhesion formation.^5^ According to several previous studies, although the mechanism of adhesion formation is not fully understood, fibroblast activity and inflammatory responses are believed to be important in the pathogenesis of adhesion formation.

The inflammatory response is a complex pathophysiological process including many chemical and cytokine mediators that cause extravascular plasma leakage (as a consequence of increased vascular permeability) and the formation of fibrin. This leads to the formation of serosanguineous exudate, which in turn initiates adhesion formation.^16^ Fibrin provides a framework for fibroblast proliferation, the synthesis of collagen and adhesion formation.^18^

Increase in reactive oxygen species (ROS) after endothelial tissue damage, which occurs during open surgery, may play a role in postoperative adhesion formation. Evidence has shown that ROS scavengers could reduce adhesion formation in animal models.^16^,^21^ Subsequently, if these initial adhesions are not lysed, they are organised into fibrous adhesions by activated fibroblasts. However, in a state of imbalance between fibrin deposition and dissolution, deposited fibrin may persist and fibrinous adhesions may develop.^16^,^21^

Unfortunately, despite continuous advances and research, an ideal method and material to decrease postoperative pericardial adhesion formation have not been found. A number of antiadhesive interventions have been developed and many have been tested clinically and experimentally in cardiac applications. Various methods and agents have been used with controversial results.^22^

Some studies have focused primarily on substitutes (autogenous, heterogenous and synthetic) providing a barrier between the epicardium and pericardium or overlying sternum, while other work has evaluated the ability of a variety of pharmacological agents to decrease or prevent pericardial adhesion formation after cardiac surgery.^16^,^23^-^25^ Anti-inflammatory drugs, antibiotics and topical application of fibrinolytic agents have also been shown to decrease pericardial adhesion formation.^16^,^26^,^27^

ABS is a herbal extract attained from five different plants: *Thymus vulgaris* (thyme), *Glycyrrhiza glabra* (licorice), *Vitis vinifera* (unriped grape), *Alpinia officinarum* (galangal) and *Urtica dioica* (stinging nettle). It has been folklorically used in traditional Turkish medicinal practice. ABS represents an alternative treatment modality for many kinds of bleeding that are resistant to conventional methods.

Today, topical ABS is used and has provided positive results in spontaneous or secondary bleeding (gastrointestinal, orthopedic, nasal, dermal) due to body injuries, traumas, and minor or major surgical interventions and wound healing.^28^ Besides its homeostatic activity, Kocak *et al.*^29^ reported that Ankaferd might also have anti-inflammatory effects.^7^ Tests have demonstrated its safety, efficacy, sterility and non-toxicity for external usage.^8^,^30^

Al *et al.*^7^ showed that Ankaferd was not efficient in reducing postoperative intra-abdominal adhesions. Conversely, Cömert *et al.*^8^ reported that there was less intra-peritoneal adhesion formation in the Ankaferd than in the control group. However, the safety and non-toxicity of Ankaferd for intra-pericardial usage and the effects of Ankaferd on postoperative pericardial adhesion have yet to be assessed.

In the present study, fibrosis score measurements showed no statistically significant difference between the Ankaferd and control groups (*p* = 0.234). However when the groups were compared according to the prevalence of fibrosis, there were statistically significant differences between the groups (*p* = 0.028), and fibrosis scores were significantly higher in the Ankaferd group. The results of our study showed that topical application of Ankaferd could increase pericardial adhesion after abrasive injury of the epicardial surface in a rabbit model.

In addition to the pericardial adhesion and visibility of coronary vessels score, histological evaluation was used to evaluate the effect of Ankaferd on inflammation and fibrosis in the rabbit model. However, there were no statistically significant differences between the groups in terms of inflammatory scores and degree of inflammation (*p* = 0.382, *p* = 0.220, respectively). Hence, efficacy of Ankaferd on inflammation was not observed in the histological evaluation.

## Conclusion

We applied Ankaferd to try and reduce postoperative pericardial adhesion in an experimental rabbit model. The use of Ankaferd increased the adhesion and fibrosis scores. However, its efficacy on inflammation was not demonstrated. Further studies with Ankaferd are necessary to evaluate its efficacy in prevention of adhesion formation in cardiac surgery.
